# Quantum integration in swin transformer mitigates overfitting in breast cancer screening

**DOI:** 10.1038/s41598-025-17075-1

**Published:** 2025-08-27

**Authors:** Zongyu Xie, Xiaoguang Yang, Shuni Zhang, Jingru Yang, Yun Zhu, Aoqi Zhang, Haitao Sun, Qun Dai, Lei Li, Hongde Liu, Wenlong Ming, Menghan Dou

**Affiliations:** 1 Department of Radiology, The First Affiliated Hospital of Bengbu Medical University, Bengbu, 233004 China; 2 Origin Quantum Computing Technology (Hefei) Co., Ltd., Hefei, 230088 China; 3 Department of Radiology, Zhongshan Hospital, Fudan University, Shanghai, 200030 China; 4 State Key Laboratory of Digital Medical Engineering, School of Biological Science & Medical Engineering, Southeast University, Nanjing, 210096 China; 5 Jiangsu Key Laboratory of Intelligent Medical Image Computing, School of Artificial Intelligence, Nanjing University of Information Science and Technology, Nanjing, 210044 China; 6 Hefei Benyuan Quantum Computing and Data Medicine Institute, Hefei, 230088 China

**Keywords:** Breast cancer, Digital mammography, Artificial intelligence, Quantum computing, Quantum information, Breast cancer, Computer science

## Abstract

To explore the potential of quantum computing in advancing transformer-based deep learning models for breast cancer screening, this study introduces the Quantum-Enhanced Swin Transformer (QEST). This model integrates a Variational Quantum Circuit (VQC) to replace the fully connected layer responsible for classification in the Swin Transformer architecture. In simulations, QEST exhibited competitive accuracy and generalization performance compared to the original Swin Transformer, while also demonstrating an effect in mitigating overfitting. Specifically, in 16-qubit simulations, the VQC reduced the parameter count by 62.5% compared with the replaced fully connected layer and improved the Balanced Accuracy (BACC) by 3.62% in external validation. Furthermore, validation experiments conducted on an actual quantum computer have corroborated the effectiveness of QEST.

## Introduction

Breast cancer remains one of the most significant health risks for women, with the highest rates of occurrence and mortality from cancer worldwide among females^1^. Early detection through breast cancer screening is crucial and has been shown to significantly reduce breast cancer-specific mortality^2^. Full-field digital mammography (FFDM) is the most widely used technique in clinical breast cancer screening due to its safety, convenience, low cost, and the absence of injected agents, which are known to cause adverse effects in some patients^3^.

With the rapid development of computer science in the past 10 years, artificial intelligence (AI), represented by machine learning (ML) and deep learning (DL)—has been increasingly applied in the analysis of medical images^4^. Simultaneously, ML-integrated radiomics has gained popularity since 2016. Compared to traditional statistical methods, ML can extract valuable insights from big data. However, ML-based radiomics relies on manually designed features, which limits its generalizability across diverse datasets. In contrast, DL methods automatically extract rich and complex features, making them more adaptable to data variations. Nevertheless, as networks become increasingly sophisticated and medical data volumes grow exponentially, the computational time and resource demands for training and inferring deep learning models pose significant challenges to traditional computing systems.

In 1980, Paul Benioff and Yuri Manin independently put forward early ideas on quantum computing. Building on this, Richard Feynman and David Deutsch later refined its core concepts and theoretical framework. Similar to classic bits, a quantum bit (qubit) also has two possible states represented in Dirac notation as ${|{0}\rangle }$ and ${|{1}\rangle }$. However, a key difference is that qubits can exist in a linear combination of two states before measurement, known as superposition state, which can be expressed as ${|{\psi }\rangle } = \alpha {|{0}\rangle } + \beta {|{1}\rangle }$. Only when a measurement is performed does the qubit collapse to either ${|{0}\rangle }$ or ${|{1}\rangle }$ with probabilities of $\Vert \alpha \Vert ^2$ and $\Vert \beta \Vert ^2$, with probabilities determined by the magnitudes of $\alpha$ and $\beta$. Additionally, through quantum entanglement – another important property of qubits – $2^n$ classical bits can be represented by *n* qubits. The superposition characteristics enable parallel computing, requiring significantly less computational resources compared to classical computing. Recent advances have demonstrated that, for certain specific problems, quantum computers can be far more efficient than classical computers^5^^6^. Recent advances in quantum computers have ushered in the so-called noisy intermediate-scale quantum (NISQ) era^7^. In the NISQ era, quantum computers can be used in real-world applications, albeit with constrained capabilities and accuracy.

Quantum machine learning(QML) is an emerging interdisciplinary field that integrates quantum computing and traditional machine learning. Variational Quantum circuit(VQC) based QML algorithms such as Quantum Support Vector Machine(QSVM)^8–11^, Quantum Convolution Neural network(QCNN)^12–16^, and Quantum Neural Network(QNN)^17,18^ were used for diagnostic tasks. However, limited by the current constraints of quantum computing, they can only handle low-dimensional data. To address this issue, there are currently two approaches. The first is to extract the features from images^8–11^ or directly perform dimensionality reduction on images^12,18^ with classical methods to lowering the data dimension, and use QML to handle low-dimensional data. The second is to embed QNNs as modules into deep learning^13–17,19–22^, which is called hybrid quantum-classical neural network(HQCNN). Those modules including Quantum Convolution^13–16^, Quantum Pooling^19^, Quantum Self-Attention Mechanisms^20^, Quantum Classifier^17,18,21,22^, etc., and Quantum Classifier enables quantum transfer learning, which is transferring knowledge from pretrained models into HQCNN^17,21,22^.

Mari et al.^23^ first proposed the quantum transfer learning paradigm, and Azevedo et al.^17^ first introduced it in breast cancer detection. Azevedo’s work indicates that quantum transfer learning improves the model’s performance, but the mechanism behind it remains unclear. Therefore, we **hypothesize** that the integration of variational quantum circuits, enabled by quantum entanglement and superposition, can result in fewer parameters compared to classical fully connected layers while maintaining or enhancing the performance of deep learning classification models. We further **hypothesize** that the mechanism underlying such maintained or enhanced performance is that the integration of variational quantum circuits can mitigate the overfitting problem in deep learning classification models.

This study makes the following contributions: (a) We compared 4 ML-based radiomics models, the Swin Transformer model, and the QEST model on breast cancer screening performance. To the best of our knowledge, this is the first study to compare ML-based radiomics, DL, and quantum-integrated DL. (b) QEST demonstrated performance comparable to the classic Swin Transformer, while the designed VQC requires only *O*(*KN*) parameters, whereas a classical linear layer needs $O(N^2)$ parameters. Visualizations using t-SNE and PCA clearly highlight the unique effect of the designed VQC. (c) Our experiments with 8 and 16 qubits were conducted on a 72-qubit real quantum computer, representing the study with the largest qubit scale ever used in breast cancer screening to date.

## Methods

### Data preparation

**Figure 1:**
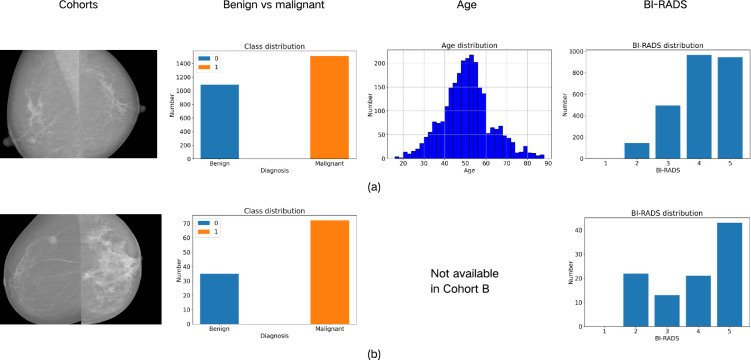
The data distribution of this study. (**a**) Data distribution of cohort A. (**b**) Data distribution of cohort B.

The distribution of data used in this study was depicted in Fig. 1. Cohort A consists of patients who underwent FFDM examinations in China between January 2019 and August 2021. A total of 2,601 cases from 1,525 patients with both biopsy results and region of interest (ROI) annotations were included in this study, while other cases were excluded. Cohort B was collected from the INbreast database^24^, an open available dataset obtained from the Breast Centre in CHSJ, Porto between April 2008 to July 2010. A total of 107 cases with ROI annotations were included in this study, with other cases excluded.

Cohort A was divided into three subsets based on the timing of data acquisition, a process referred to as “temporal validation”^25^. The split ratio followed 7:1:2^26^: specifically, the first 70% of the data was allocated to the training set for model training; the middle 10% formed the validation set, which was used to adjust hyperparameters for models with trainable parameters; and the final 20% constituted the test set, utilized for internal evaluation and validation on a real quantum computer. Cohort B was exclusively employed for external evaluation to serve multi-center validation purposes. The distributions of Cohort A and Cohort B are visualized in Fig. 1. For quantum computer validation, 96 samples were randomly selected from the test set.

Cohort A is in nearly raw raster data (NRRD) format, while Cohort B is in digital imaging and communications in medicine (DICOM) format. To address discrepancies in data formats, all data were converted to Portable Network Graphics (PNG) format using min-max normalization, scaling the pixel value to integers between 0 and 255. For the radiomics models, initial features were extracted from the normalized images with the guidance of ROI masks using pyradiomics^27^. Most features adhere to the definitions outlined by the Imaging Biomarker Standardization Initiative (IBSI)^28^. For DL and quantum-integrated DL, ROIs identified by the mask annotations were cropped from the full images as the input of the models.

### Radiomics models

This study included four machine learning models as radiomics classifiers: Support Vector Machine (SVM)^29^, a kernel-based model that uses a kernel function to approximate the similarity of sample pairs in high-dimensional spaces. In this study, the Radial Basis Function (RBF) kernel was used with SVM. Logistic Regression (LR)^30^, a simple yet effective linear model. K-Nearest Neighbor (KNN)^31^ a distance-based model. Multi-Layer Perceptron (MLP)^32^, a neural network-based model.

The initial data dimension was 851, as described in the preprocessing section. The Least Absolute Shrinkage and Selection Operator (Lasso)^33^ was applied, reducing the dimension to 58, and Spearman Correlation Threshold(SRT) was then used to further reduce the dimension to 8 and 16, respectively.

### Swin transformer

Swin Transformer^34^ is an improved version of the Vision Transformer^35^ model. It incorporates a hierarchical structure and shifted window-based self-attention mechanism, enabling the model to effectively handle high-resolution images, a common characteristic of medical imaging data. The hierarchical representation allows the model to capture both local and global features, which is crucial for accurately diagnosing lesions of various scales within an image. In this study, Swin B, a medium-scale Swin Transformer model, was selected as the feature extractor, and a multilayer perceptron(MLP) was used as the classic classifier.

A transfer learning scheme was adopted to alleviate the shortage of training data, where the weights of the feature extractor were initialized by weights pre-trained on the ImageNet dataset^36^, and then fine-tuned on the training set. The output dimension of Swin B was rectified to be consistent with the input dimension of the classifier. The input images were first preprocessed as described in the data preprocess section, and then resized to 224 $\times$ 224 pixels to accommodate the input size of Swin B.

A data augmentation process was performed after the resizing, which is consisted of horizontal and vertical flips at 50% probability, random rotation of up to 10 degrees, and color jitter. Finally, the data were normalized according to the mean and standard deviation of ImageNet dataset. Except for the preprocess, all the subsequent processes were performed in an online manner. Cross entropy loss with the label smooth was selected as the loss function. The models were trained for 80 epochs with a learning rate of 0.0004. The losses values all showed convergence at the 80th epoch on the validation set. The general framework of the swin transformer is shown in Fig. 3b.

### Quantum embedding

Quantum embedding is the process to encode classical data $\vec {x}$ into quantum states ${|{x}\rangle }$. Angle embedding, Amplitude embedding and Basis Embedding were discussed in this study. Angle embedding encodes $N$ features into the rotation angles of $n$ qubits, where $N\le n$. Amplitude embedding encodes $2^n$ features into the amplitude vector of $n$ qubits. Basis embedding encodes $n$ binary features into a basis state of $n$ qubits.

**Table 1:** Comparison of three kinds of quantum embedding.

Encoding methods	Number of qubits	Circuit depth	Need controlled gates	Feature types
Angle embedding	$\le N$	*O*(1)	No	Real number
Amplitude embedding	$\log _2 N$	*O*(*N*)	Yes	Real number
Basis embedding	*N*	*O*(1)	Yes	Binary number

As shown in Table 1, where $N$ represent the number features, Basis Embedding only support binary numbers, while features in Deep Neural Network (DNN) are continued real numbers, which make it unsuitable for our experiment. Amplitude embedding is efficient in qubit number, which it requires $O(N)$ depth and a great amount of controlled gates, which make it improper to perform on real quantum computer in NISQ era. Therefore, we perform comparison experiment for angle embedding and amplitude embedding on simulators rather than real quantum computers. On the contrary, Angle embedding requires $O(1)$ depth, and no controlled gates are involved, which make it highly suitable for experiments on real quantum computers.

#### Angle embedding

The angle range of quantum gates is real numbers from $-\pi /2$ to $+\pi /2$, while the value range of classical features is real numbers from $-\infty$ to $+\infty$. Therefore, it is necessary to first adjust the value range of the features through the arctangent function to achieve a one-to-one correspondence between classical features and quantum gate angles.

1 $$\begin{aligned} x_i^{'} = \arctan {x_i} \end{aligned}$$

Each element $x_i$ of $\vec {x}$ was normalized to the range $[-\pi /2, \pi /2]$ using Eq. 1. The normalized value was then encoded as the rotation angle for Y-gates, while the square of this normalized value was further encoded as the rotation angle for Z-gates- a step aimed at enhancing the representational capacity, as detailed in Eq. 2.

2 $$\begin{aligned} {|{\psi _x}\rangle }=\bigotimes _{i=1}^{N} RZ((x_i^{'})^2)(RY(x_i^{'})(\frac{1}{\sqrt{2}}({|{0}\rangle }+{|{1}\rangle }))) \end{aligned}$$

#### Amplitude embedding

Amplitude embedding leverages the amplitudes of quantum states to encode classical features, enabling an exponential increase in data capacity. Unlike angle embedding, which encodes features to rotation angles, amplitude embedding represents an $N$-dimensional classical vector $\vec {x}$ as the amplitudes of $n = \lceil \log _2 N \rceil$ qubits, where each basis state corresponds to an element of the vector.

However, raw classical vectors $\vec {x}$ must first satisfy the quantum state normalization condition $\sum _{i=1}^{N} |x_i|^2 = 1$. For arbitrary classical data, this requires pre-processing through $L^2$-normalization:

3 $$\begin{aligned} x_i' = \frac{x_i}{\Vert \vec {x}\Vert _2} \quad \text {where} \quad \Vert \vec {x}\Vert _2 = \sqrt{\sum _{i=1}^{N} |x_i|^2} \end{aligned}$$

Each element $x_i$ of $\vec {x}$ is normalized using Eq. 3, transforming the classical features into a valid quantum state ${|{\psi _x}\rangle }$:

4 $$\begin{aligned} {|{\psi _x}\rangle } = \sum _{i=0}^{N-1} x_i' {|{i}\rangle } \end{aligned}$$

This encoding achieves an exponential advantage in data capacity, as $n$ qubits can represent $2^n$ dimensions. However, implementation of Eq. 4 often requires non-trivial quantum circuits, which lead to $O(N)$ circuit depth.

###  Variational quantum circuits (VQC)

**Figure 2:**
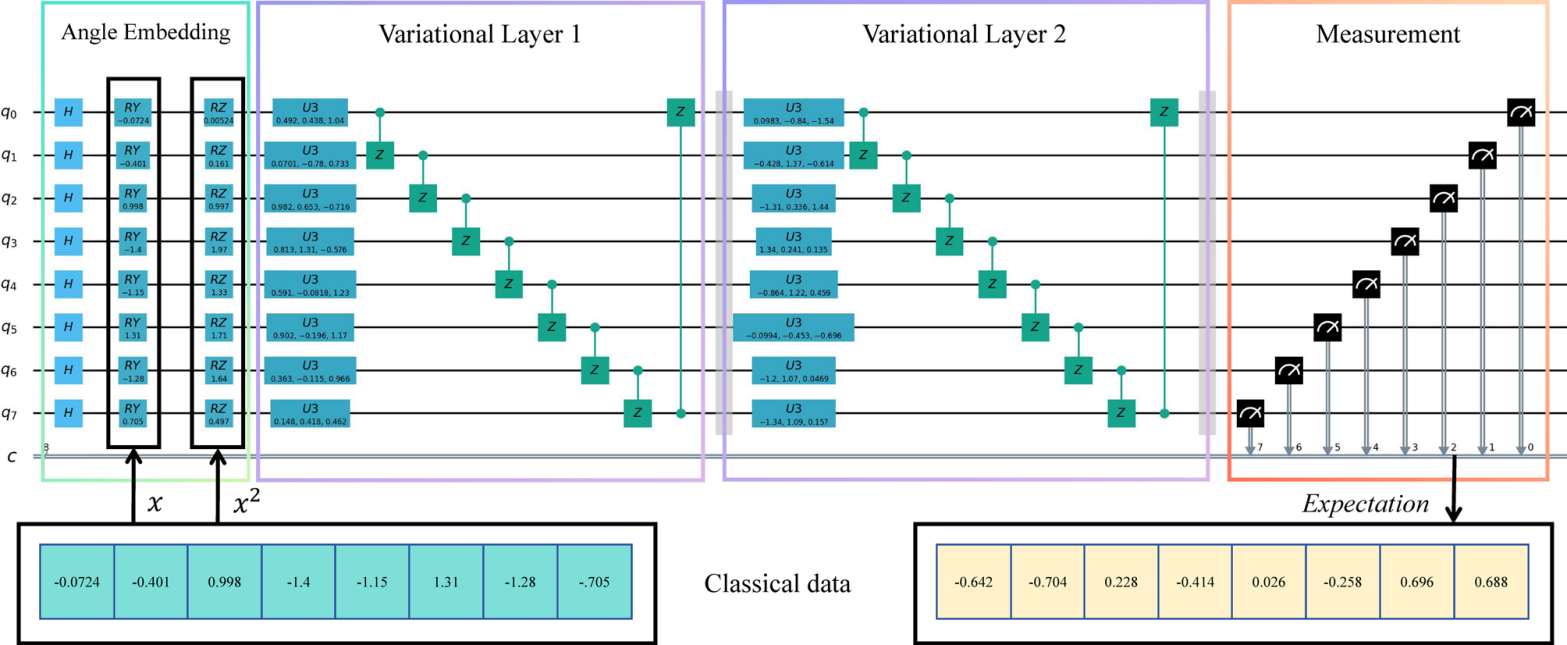
The illustration of the designed VQC in 8 qubits case.

VQC is a specific implementation of QNN, and in some literatures, the two terms can be used interchangeably. Similar to classical neural networks, VQC features trainable parameters. In this study, a VQC structure inspired by the circuit-centric quantum classifier^38^ was adopted in most experiments. The designed VQC retains the strong entangling architecture of the circuit-centric quantum classifier but replaces amplitude embedding with angle embedding (as depicted in Fig. 2), with the rationale discussed in the quantum embedding section. This circuit is shallow and strongly entangling, making it suitable for classification tasks^38^. Furthermore, by directly utilizing the basic gates of the target quantum computer, the circuit is made even shallower, since gate decomposition is avoided, making it more suitable for implementation on real quantum computers.

The VQC comprises three modules: (1) Embedding module(see Eqs. 1,2); (2) Variational module $U_f$(see Eq. 5); (3) Measurement module (see Eq. 6). In Eq. 6, the expectation value $\langle {\hat{Z}} \rangle$ is obtained by measuring the composite quantum state ${|{y}\rangle }$ in the computational basis. Here $\vec{\theta}_i$ denotes the trainable parameters and *N* specifies the number of qubits. The whole process is depicted in Fig. 2.

5 $$\begin{aligned} {|{\psi _y}\rangle }= & U_f(\vec {\theta }_1)U_f(\vec {\theta }_2){|{\psi _x}\rangle } \end{aligned}$$

6 $$\begin{aligned} \vec {y}= & {\langle {\psi _y}|}Z^{\otimes N}{|{\psi _y}\rangle } \end{aligned}$$

Qubits were first initialized at zero states, and then prepared into uniform superposition states through Hadamard gates. Two consecutive variational layers were used as the variational layers. Within the variational structure, controlled Z-gates were used for quantum entanglement, and U3 gates were used for learning. Measurement was performed with 1000 shots during the Monte Carlo based simulation, and the Pauli-Z expectations of the measured states constituted the output.

### Quantum-enhanced swin transformer (QEST)

**Figure 3:**
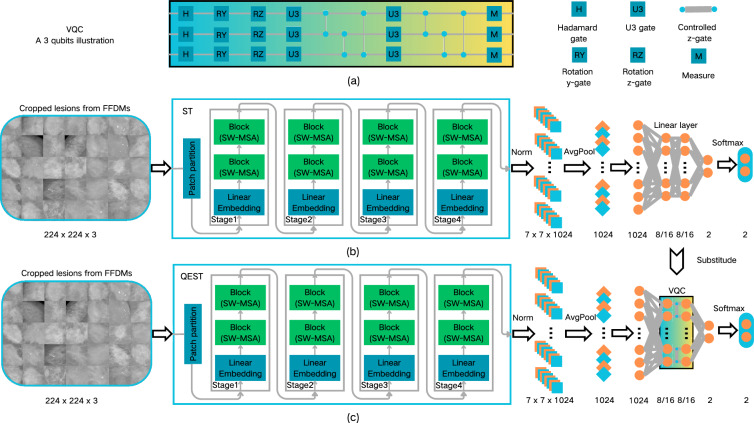
The design of the workflow for this study. (**a**) The circuit structure of the designed VQC. (**b**) The process chart of Swin Transformer. (**c**) The process chart of QEST. VQC variational quantum circuit.

QEST adopted the quantum transfer learning paradigm, where a VQC (as illustrated in Fig. 3a) is integrated at the end of the Swin Transformer to replace the fully connected (FC) layer, as shown in Fig. 3c. For comparative experiments, the output dimensions of the third-to-last layer and the input dimensions of the final linear layer in the hybrid network were set to 8 or 16, with matching dimensions. The VQC was optimized jointly with other components of the hybrid network, enabling the network to effectively learn how to prepare inputs and post-process outputs for the VQC. To address data imbalance, resampling methods were adopted to construct a balanced data loader.

In the training process, the selected optimizer was SGD, with a momentum of 0.9 and weight decay of 1e-4. A cosine annealing scheduler was adopted, and all network components were trained for 80 epochs. Specifically, two distinct learning rates were used: 0.0004 for the classical part and 0.004 for the VQC, as the VQC benefits from a larger learning rate. This is because the output range of the VQC is smaller than that of a fully connected layer.

### Performance evaluation

BACC Eq. 7, sensitivity Eq. 8, specificity Eq. 9, the receiver operating characteristic curve (ROC), and the area under ROC curve (AUC) were used as performance measurement. Models were trained on the training set, and evaluated on both the internal and external test sets respectively. Since only binary classification was studied in this paper, true positive (TP), true negative (TN), false positive (FP), and false negative (FN) values were calculated as intermediate results for the above metrics. The definitions of BACC, sensitivity, and specificity are as follows:

7 $$\begin{aligned} BACC= & \frac{1}{2}(Sensitivity + Specificity) \end{aligned}$$

8 $$\begin{aligned} Sensitivity= & \frac{TP}{TP+FN} \end{aligned}$$

9 $$\begin{aligned} Specificity= & \frac{TN}{TN+FP} \end{aligned}$$

GradCAM^39^ and GradCAM++^40^ were used to visualize the basis for the model’s judgment. Principal Component Analysis(PCA)^41^ and t-distributed Stochastic Neighbor Embedding(t-SNE)^42^ were applied to visualize the effect of the VQC and the linear layer.

### Quantum computer

The quantum computer verification experiments were conducted on a 72-qubit superconducting quantum computer “Origin Wukong”. The quantum chip “Wukong Core” used by Origin Wukong has a total of 198 qubits, including 72 working bits and 126 coupler qubits. The energy relaxation time T1 is 14.51$\upmu$s, and the phase relaxation time T2 is 1.84$\upmu$s. The basic logic gates are U3 and CZ gates, the maximum number of layers is 500, and the maximum number of measurements is 65535.

When physical qubits have constrained connectivity, logical quantum circuits must undergo a mapping process onto physical hardware, known as the Qubit Mapping Problem (QMP). As a critical step in quantum circuit compilation, QMP requires the insertion of SWAP gates to reposition qubits and thus satisfy hardware connectivity constraints. Notably, its decision version is NP-complete. For large-scale circuits or complex architectures, automatic mapping methods often exhibit inefficiency: they introduce an excessive number of SWAP gates, which in turn increase circuit depth and elevate error rates^46–48^.

**Figure 4:**
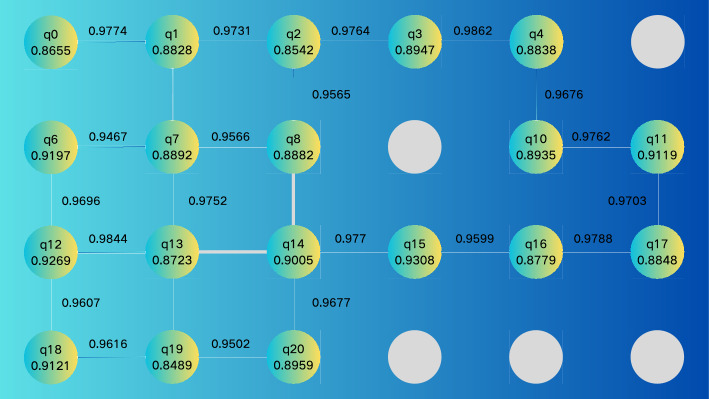
The topology of the used qubits and their entanglements. The indices of physical qubits and their single-qubit readout fidelities are displayed within the corresponding nodes, while the fidelities of controlled-Z gates for entangled qubit pairs are indicated above the lines connecting them.

To address this, we manually selected a physical qubit topology that precisely matches our quantum circuit design, thereby eliminating the need for SWAP gates. Additionally, we pre-screened out single and two-qubit connections with low fidelity. This approach ensures that the chosen physical qubits exhibit high fidelity in both single and two-qubit operations, thereby securing superior performance on real quantum devices. The topology of the qubits utilized in this study is illustrated in Fig. 4, where the connectivity aligns perfectly with our quantum circuit design, further obviating the requirement for extra SWAP gates.

Matrix-free Measurement Mitigation (M3)^49^ was adopted as the error mitigation method. M3 mainly consists of three steps, and it is implemented on the noise-affected results. First, find the physical qubits that the virtual qubits are mapped to. Next, calibrate the physical qubits to obtain their measurement noise distribution. Finally, correct the counting results obtained from the circuit operation.

## Results

### Encoding methods and hyperparameters choice

This section includes experiments on encoding methods, the number of variational layers in VQC, different gate configurations, and optimization methods. Specifically, angle embedding and amplitude embedding, 1-5 variational layers, CNOT, RY and RZ, and SGD, Adam and AdamW were included. The results are shown in Table 2.

**Table 2:** The performance comparison of different encoding methods and different hyperparameter configurations.

	ACC	BACC	AUC	SEN	SPE
Encoding					
Angle embedding	0.902	0.904	0.960	0.933	0.865
Amplitude embedding	0.885	0.888	0.956	0.927	0.837
Number of variational layer					
1	0.898	0.901	0.961	0.935	0.855
2	0.902	0.904	0.960	0.933	0.865
3	0.900	0.902	0.958	0.932	0.862
4	0.900	0.902	0.959	0.932	0.862
5	0.896	0.898	0.955	0.932	0.855
Variational gates					
U3+CZ	0.902	0.904	0.960	0.933	0.865
U3+CNOT	0.887	0.892	0.953	0.940	0.829
RY+CZ	0.894	0.896	0.957	0.926	0.857
Optimizer					
SGD	0.902	0.904	0.960	0.933	0.865
Adam	0.865	0.872	0.941	0.931	0.799
AdamW	0.875	0.881	0.944	0.936	0.813

It should be noted that amplitude embedding requires only 4 qubits, whereas angle embedding requires 16 qubits. However, amplitude embedding exhibits inferior performance compared to angle embedding and demands a deeper circuit depth, which is why it was not adopted in the real quantum computer setup. Regarding the number of variational layers, it has little impact on the model performance: performance peaks at 2–4 layers and slightly declines when reaching 5 layers. Additionally, the gate combination of U3 + CZ yields relatively favorable results. In terms of optimization, SGD shows significant advantages in optimizing the QEST model. In contrast, Adam and AdamW failed during training when performing full network optimization; thus, we adopted a strategy of fixing the weights of the backbone network. Other hyperparameters were also tuned through experiments.

###  Model comparison

#### Performance comparison

We compared four radiomics models (KNN, LR, SVM, and MLP), Swin Transformer, and QEST under the conditions of 8 and 16 features after dimensionality reduction. Since we adopted angle embedding, 8 and 16 features actually correspond to 8 qubits and 16 qubits, respectively.

**Table 3:** The performance of different assessment approaches for predicting malignant or benign breast ROI across two cohorts.

	Cohort	Approach	BACC (95% CI)	AUC (p-value)	SEN	SPE
8 features/qubits	Internal	KNN	0.824 (0.790, 0.857)	0.890 (< 0.001)	0.858	0.791
		LR	0.831 (0.798, 0.863)	0.907 (< 0.001)	0.814	0.849
		SVM	0.863 (0.833, 0.893)	0.926 (< 0.001)	0.878	0.849
		MLP	0.861 (0.829, 0.890)	0.937 (< 0.001)	0.881	0.884
		SwinTransformer	0.896(0.869, 0.922)	0.955(= 0.575)	**0.881**	0.911
		QEST	**0.898 (0.871, 0.922)**	**0.958(= 1.000)**	0.871	**0.924**
	External	KNN	0.630 (0.545, 0.716)	0.714(=0.002)	**0.917**	0.343
		LR	0.617 (0.524, 0.708)	0.763(= 0.015)	0.833	0.400
		SVM	0.666 (0.577, 0.756)	0.704(= 0.005)	0.903	0.429
		MLP	0.740 (0.650, 0.824)	0.793(= 0.098)	0.833	0.429
		SwinTransformer	0.774 (0.682, 0.859)	**0.871(= 0.686)**	0.806	0.743
		QEST	**0.788 (0.700, 0.870)**	0.864 (= 1.000)	0.833	**0.743**
16 features/qubits	Internal	KNN	0.808 (0.773, 0.841)	0.883(< 0.001)	0.851	0.764
		LR	0.843 (0.811, 0.874)	0.920 (< 0.001)	0.851	0.836
		SVM	0.858 (0.827, 0.887)	0.930 (< 0.001)	0.858	0.858
		MLP	0.858 (0.827, 0.888)	0.941 (< 0.001)	0.837	0.880
		SwinTransformer	0.898 (0.871, 0.924)	0.955(= 0.426)	0.881	0.916
		QEST	**0.904 (0.877, 0.928)**	**0.960 (= 1.000)**	**0.892**	**0.916**
	External	KNN	0.552 (0.547, 0.716)	0.697 (=0.002)	0.847	0.257
		LR	0.668 (0.524, 0.711)	0.776 (=0.027)	0.764	0.571
		SVM	0.617 (0.576, 0.755)	0.711 (=0.006)	0.833	0.400
		MLP	0.705 (0.61, 0.792)	0.802 (=0.268)	**0.903**	0.429
		SwinTransformer	0.774 (0.686, 0.857)	**0.876 (=0.345)**	0.806	0.743
		QEST	**0.802 (0.716, 0.881)**	0.860 (= 1.000)	0.861	**0.743**

**Figure 5:**
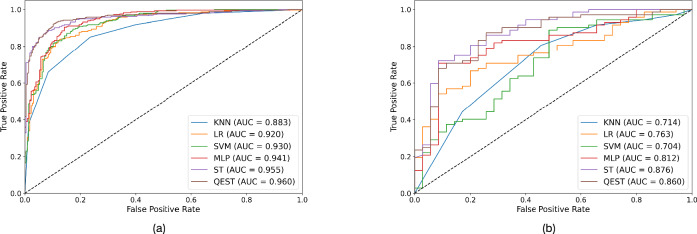
ROC curves of 6 models in 16 features case. (**a**) ROC curves on Internal validation; (**b**) ROC curves on external validation. ROC receiver operating characteristic.

As shown in Table 3, first of all, Swin Transformer and QEST have obvious advantages over other models. Secondly, compared with Swin Transformer, QEST has a more prominent advantage in BACC: it achieved a higher BACC in all experiments, with a leading margin ranging from 0.02% to 0.28%. In terms of AUC, however, QEST and Swin Transformer performed comparably. QEST achieved a higher AUC only in the external validation with 8 features and the internal validation with 16 features, while Swin Transformer achieved a higher AUC in the 8-qubit internal validation and 16-qubit internal validation. We used the Delong test to compare the differences between other models and QEST. The results showed that QEST had significant differences from the radiomics models in most cases, with most p-values less than 0.001. However, the difference between QEST and Swin Transformer was not significant, with p-values ranging from 0.345 to 0.686. Figure 5 shows the comparison of ROC curves between models, from which it can be found that Swin Transformer and QEST are highly similar, while their differences from other models are relatively obvious.

#### Loss comparison

**Figure 6:**
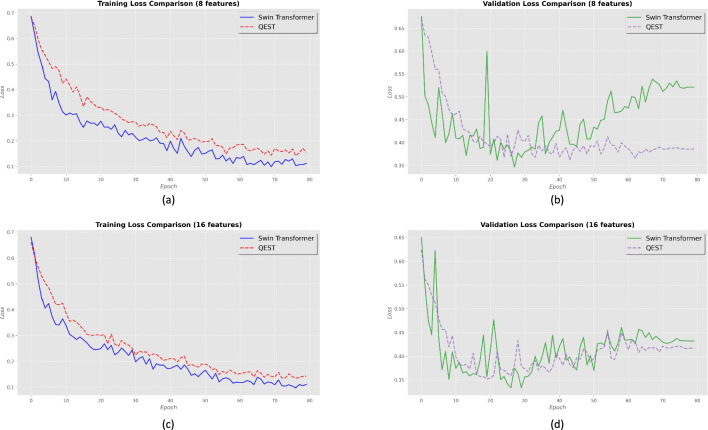
The loss comparison of Swin Transformer and QEST on training set and validation set. (**a**) The training loss comparison of Swin Transformer and QEST of 8 feature case. (**b**) The validation loss comparison of Swin Transformer and QEST of 8 feature case. (**c**) The training loss comparison of Swin Transformer and QEST of 16 feature case. (**d**) The validation loss comparison of Swin Transformer and QEST of 16 feature case.

As shown in Fig. 6, with both 8 and 16 features, the training set loss of QEST is consistently slightly higher than that of Swin Transformer, while its validation set loss fluctuates less compared with Swin Transformer and eventually converges to a lower value. Specifically, when converging on the validation set, the loss of QEST is 0.135 lower than that of Swin Transformer with 8 features, and 0.015 lower than that of Swin Transformer with 16 features. In fact, the loss of QEST, whether on the training set or the test set, is more stable than that of Swin Transformer, which indicates that the training of QEST is more stable. Additionally, the difference between the training loss and validation loss of QEST is smaller than that of Swin Transformer, showing that QEST is less prone to overfitting during training compared with the latter.

### Quantum computer validation

**Figure 7:**
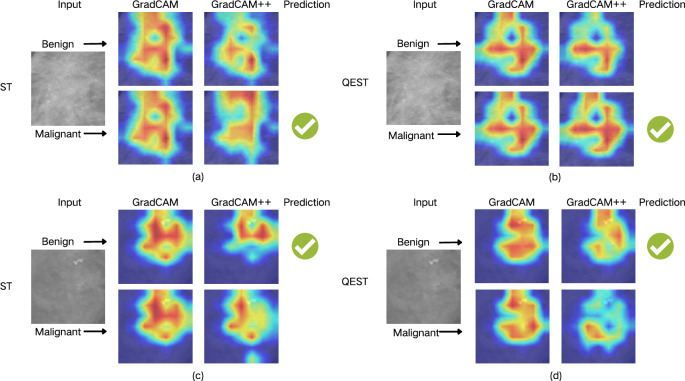
GradCAM and GradCAM++ comparison of swin transformer and QEST. (**a**,**b**) A malignant case. (**c**,**d**) A benign case with different attentions. *ST* swin transformer.

**Figure 8:**
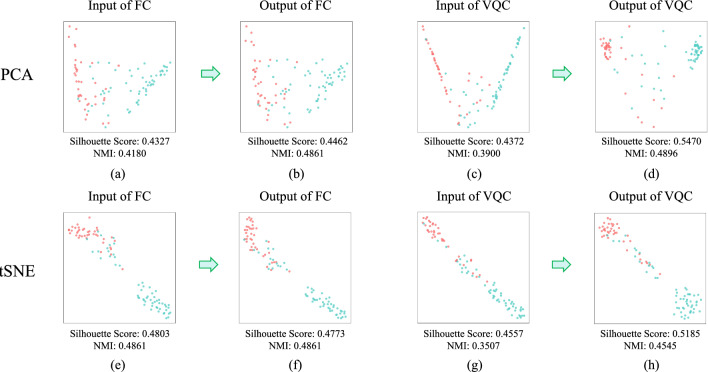
PCA and t-SNE embedding distance visualization of 16 features/qubits cases on a real quantum computer. (**a**) The PCA embedding distance visualization of the input of FC. (**b**) The PCA embedding distance visualization of the output of FC. (**c**) The PCA embedding distance visualization of the input of VQC. (**d**) The PCA embedding distance visualization of the output of VQC. (**e**) The t-SNE embedding distance visualization of the input of FC. (**f**) The t-SNE embedding distance visualization of the output of FC. (**g**) The t-SNE embedding distance visualization of the input of VQC. (**h**) The t-SNE embedding distance visualization of the output of VQC.

Table 4 demonstrates the result of QEST on quantum computer outperformed that of the simulator in 8 qubits and 16 qubits cases, indicating that the experiments conducted on the real quantum hardware achieved favorable accuracy. In Fig. 7, a heatmap is used to visualize the model’s attention. In Fig. 8, we reduced the 16-dimensional features of 96 samples to two dimensions using t-SNE and PCA respectively, and visualized the distribution of the dimensionality-reduced features.

**Table 4:** The performance of QEST on a subset of the internal test dataset which contains 96 samples using the simulator and quantum computer.

	Device	BACC	AUC	SEN	SPE
8 qubits	Simulator	0.827	0.947	0.807	0.846
	QC	0.827	0.960	0.807	0.846
16 qubits	Simulator	0.849	0.948	0.877	0.821
	QC	0.850	0.958	0.930	0.769

*QC* quantum computer.

#### Heatmap analysis

A malignant case and a benign case were randomly selected from Cohort A. Figure 7 presents the GradCAM and GradCAM++ visualization results of Swin Transformer and QEST in diagnose the two cases.

The heatmaps depict regions with different outcome correlations, where red indicates a higher contribution to malignant/benign predictions and blue indicates a lower contribution. In the gradient heatmaps, the color gradient from cold (blue) to warm (red) signifies increasing attention. For malignant cases (**a**,**b**), both models made correct predictions, but their attention regions differ significantly, as indicated by the arrows pointing to the malignant heatmaps. For benign cases (**c**,**d**), the predictions are consistent, yet their attention regions do not overlap either, with arrows highlighting the benign heatmaps. This suggests that the two models make judgments based on different image features. Such differences reflect that quantum enhancement has altered the decision-making basis of the model, making QEST and Swin Transformer “equally good but distinct.” This is conducive to improving the diagnostic performance of the ensemble model and also confirms that the VQC prompts the model to learn unique features, providing a new perspective for screening.

#### Dimensionality reduction visualization analysis

In this experiment, we calculated the Silhouette Score and Normalized Mutual Information (NMI). The Silhouette Score measures intra-cluster compactness and inter-cluster separation, while NMI-based on ground-truth labels-gauges the consistency between clustering results and real categories. These metrics were evaluated on feature data after t-SNE and PCA dimensionality reduction, respectively.

As shown in Fig. 8, the quantitative indicators reveal that feature clusters after VQC yield higher Silhouette Scores and NMI, indicating superior clustering separation compared to fully connected layer (FC). This aligns with the visualization results, verifying that the distinguishability of QEST feature distributions outperforms that of Swin Transformer. Furthermore, comparing the Silhouette Scores and NMI before and after applying the VQC and the FC, we find that the VQC plays a significant role, whereas the FC has a much smaller effect.

## Discussion

QML is an interdisciplinary field that combines quantum computing with ML. It has been demonstrated that well-designed quantum neural networks can outperform classical neural networks due to their higher effective dimensionality and faster training capabilities^50^. Moreover, a VQC requires *O*(*KN*) parameters, in contrast to the $O(N^2)$ parameters needed for a classical linear layer. In fact, $K$ is an experimentally determined constant, and in our experiment, setting $K$ to 2 is sufficient for 8-qubit and 16 qubit circuits.

In our experiments, QEST reduced the number of parameters in the classifier by replacing one classical fully connected layer in the Swin Transformer’s classifier module, while achieving performance comparable to that of the original Swin Transformer. This outcome directly validates our first hypothesis. Although the Delong test indicates no significant difference in AUC values between the two models, a detailed loss analysis reveals that the Swin Transformer exhibited obvious overfitting during training, whereas QEST significantly alleviated this issue. This finding confirms our second hypothesis: VQC can enhance the performance of deep learning models by mitigating overfitting. The underlying mechanism may stem from the theoretical advantages of VQC, specifically their ability to maintain high expressiveness with fewer parameters^50^ and their lower requirement for training data to achieve effective generalization^53^. Through dimensionality reduction visualization analysis, it is found that the integration of VQC has a significant impact on the representation of the model. Both qualitative views and quantitative indicators indicate that the embedding of VQC significantly improves the distinguishability of features from different categories, while the linear layer has no obvious impact on the representation. This shows that integrating VQC into deep learning may help improve the performance of deep learning classifiers and enable model to learn better representations. Gradient heatmaps confirmed that quantum enhancement significantly altered the basis for model judgment, creating a comparable but distinct model. This distinction is advantageous for building ensemble models^51,52^. Additionally, experiments conducted on a real quantum computer confirmed the feasibility of leveraging current quantum technology to improve breast cancer screening performance.

Our work is inspired by the work of Azevedo et al.^17^. They applied quantum transfer learning in breast cancer diagnosis and achieved better results than the baselines. However, they did not explain the mechanism behind this superior performance, and did not include traditional radiomics and transformer-based models in their study. Additionally, their experiments only used 4 qubits, and did not expand to larger number of qubits. In this study, we developed a QEST model, which was built by replacing a linear layer in the Swin Transformer classifier to our VQC, and compare the performance of 4 ML-based radiomics models, including SVM, LR, KNN, and MLP, Swin Transformer and QEST. The experiments size was expanded to 8-qubit and 16-qubit. QEST demonstrated superior performance across most of evaluation metrics compared to other models while using fewer parameters than the Swin Transformer Classifier. This is attributed to quantum entanglement, which breaks the fixed number of parameters needed to construct a full connected layer. We further analysed the mechanism behind the enhancement. QEST can be applied to high-resolution medical imaging modalities, such as 3D breast MRI, since the VQC is integrated in the end of the model.

While this study is still limited, the first limitation lies in the scale of qubits, which is constrained by hardware. Current quantum computers face multiple challenges, including limited qubit counts, low entanglement fidelity, high resource requirements, and dependencies on classical-quantum hybrid architectures. As a result, scalability experiments were restricted to 16 qubits, and the depth of the VQC had to be kept shallow, thus limiting the number of variational layers. Moreover, if more qubits were to be used, it would likely lead to an increase in circuit depth, which in turn could reduce the computational accuracy of VQC. Additionally, manual qubit mapping would become extremely difficult in such cases, making it necessary for future research to develop better automatic mapping methods to fully address this issue.

The second limitation is that this study did not incorporate VQC integration in feature extraction. Although numerous studies have demonstrated the effectiveness of applying VQC to feature extraction, the number of operations involved could be very large. This would pose challenges in terms of the consumption of real quantum machine time and the communication between classical and quantum computations. However, this issue is expected to be resolved with the maturity of quantum computers and quantum operating systems in the future.

## Conclusion

In conclusion, we proposed QEST, a quantum-enhanced Swin Transformer. This model has demonstrated its effectiveness in breast cancer screening on real quantum computers under both 8-qubit and 16-qubit configurations, achieving performance comparable to that of the classical Swin Transformer. Notably, the integrated VQC requires only *O*(*KN*) parameters, in contrast to the $O(N^2)$ parameters demanded by classical fully connected layers. Experimental results further revealed that the enhancement mechanism of VQC intergration in deep learning models is associated with its ability to mitigate overfitting. Thus, this advantage is expected to keep in other modalities such as MRI and CT, and larger numbers of qubits.

## Data Availability

The datasets used and analyzed during the current study available from the corresponding author on reasonable request.
